# Influence of Reinforcement Architecture on Behavior of Flax/PLA Green Composites under Low-Velocity Impact

**DOI:** 10.3390/ma17122958

**Published:** 2024-06-17

**Authors:** Samuel Charca, Liu Jiao-Wang, Carlos Santiuste

**Affiliations:** 1 Department of Mechanical Engineering, University of Engineering and Technology, Barranco 15063, Peru; samuel.charca@upr.edu; 2 Continuum Mechanics and Structural Analysis Department, Universidad Carlos III de Madrid, 28903 Madrid, Spain; lwang@pa.uc3m.es

**Keywords:** biocomposites, weave pattern, low-velocity, impact, compression after impact

## Abstract

The main goal of this study is the comparison of different reinforcement architectures on the low-velocity impact behavior of green composites. The study includes the comparison of unidirectional, basket weave, and twill weave flax/PLA composites, they are subjected to unidirectional tensile tests, drop-weight impact tests, and after-impact compression tests. Results show that the unidirectional composite demonstrates superior tensile strength and initial modulus due to reduced fiber crimp, while basket weave exhibits the highest energy absorption capability and strain capacity attributed to its higher fiber–weight ratio and fiber crimp. Unidirectional composite also shows a larger impacted damage area compared to basket weave and twill weave, attributed to its internal architecture. Residual compressive strength across all composites decreased by 40% compared to the reference sample. However, the reduction in stiffness after impact was different, UD/PLA composite stiffness was reduced by 30% while the reduction in BW/PLA and T/PLA composites was about 20%.

## 1. Introduction

In recent years, natural fiber-reinforced composites (NFRP) have attracted widespread interest from academics because of their lightweight, affordability, sustainability, and biodegradability. Its development continues, with the hope of achieving high performance and creating reliable standards to expand the application of this type of composite beyond the automotive field to other fields such as construction and consumer goods [1].

Continuous natural fibers present excellent mechanical properties, making them ideal for fabricating fiber-reinforced polymer composites. There are different techniques to transform single fibers into fabrics, such as knitting and weaving, and according to Aisyah [2], woven fiber-reinforced composites have good stiffness, strength, and dimensional stability. Nonetheless, different factors can significantly affect the performance of fiber-reinforced composites. For example, depending on the weaving technique, the composite becomes more conducive to one operating condition or another, as different weave patterns can provide different levels of structural strength and energy absorption capacity.

On the other hand, different weaving patterns lead to different fiber crimps, which are defined as curls or waves along the fiber length. The presence of crimp in the fabric is beneficial because it improves fiber cohesion [3,4,5], however, a high fiber crimp level also implies relatively lower mechanical properties because during loading, the initial load is consumed by fiber stretching thus resulting in lower strength and higher strain concentration [6,7].

Ahmed et al. [8] examined unidirectional, satin, and plain weave patterns of E-glass/polyester composite under tensile and impact tests. The comparison analysis showed that the unidirectional fiber-reinforced composite had better tensile and impact properties than the other sample structures due to a lower yarn crimp. However, despite the remarkably better longitudinal tensile properties of unidirectional FRP composites, they face lower shear and transverse tensile strengths than woven FRPs [9]. Comprehensive studies on the micro- and macroscopic properties of FRP in the open literature have concluded that the choice of different reinforcement architecture depends on the desired application of the further composite.

Umair et al. [10] have studied the effect of fabric weaving patterns and the influence of the glass microsphere reinforcement on the low-velocity impact (LVI) response of hemp/green epoxy composites. They found that the 2/2 matt weave hemp/epoxy composite showed the lowest peak force and relatively high energy absorption resulting thus to higher damage area due to the lowest percentage of crimp and higher compactness of the woven structure. In comparison, the satin weave composite showed the highest impact force and the lowest displacement because of the more extended yarn float structure.

Habib et al. [11] analyzed three types of NFRP composites (jute/hemp/flax) and analyzed the influence of the weaving pattern on the short-beam shear (SBS), LVI, and compression after impact (CAI) behavior. They concluded that the knitted commingled sample presented better interlaminar shear and CAI behavior for the three composites, and the woven one exhibited higher impact resistance than the knitted and woven commingled samples thanks to its interlacement pattern.

However, there is a lack of studies about the influence of reinforcement architecture on the behavior of green composites (with both fibers and matrix biodegradable and manufactured from vegetal resources) under low-velocity impact. The present study comparatively analyzes the impact behavior and residual compression performance of green composites manufactured with a PLA matrix reinforced with three different flax fiber configurations: unidirectional, basket weave, and twill weave.

## 2. Materials and Manufacturing

The composite matrix was the polylactic acid (PLA) PLI005 acquired from NaturePlast, Mondeville, France in form of pellets. The properties are similar to other PLA products: Young modulus of 3.5 GPa, tensile strength of 50 MPa, density of 1.24 g/cm^3^, melting temperature of 175 °C, and heat deflection temperature of 60 °C. Three different types of flax configurations were used as the reinforcement in this work to cover the most used reinforcement configuration in low-velocity impact tests:

- Basket weave (BW) flax fabric acquired from a local supplier, in Madrid, Spain (areal density of 463.3 g/m^2^; thickness of 0.94 mm).
- Unidirectional (UD) flax fabric purchased from EcoTechnilin, Valliquerville, France (areal density of 180 g/m^2^; thickness of 0.36 mm).
- Twill weave 2-2 (T) flax fabric purchased from EcoTechnilin, Valliquerville, France (areal density of 360 g/m^2^; thickness of 0.83 mm).

Before manufacturing the biocomposite laminate, thin sheets of polylactic acid (PLA) were made using two heating plates under 2 MPa of pressure and 180 °C; thereafter, the flax fabrics were dried in an oven at 60 °C for 30 min. The thicknesses of the three composites were different because the objective was to produce composites with similar areal densities because it is the main variable used in impact problems, see Table 1. The hand-laid-up method was used to place the PLA and natural fiber plies. Different stacking sequences and compression conditions were taken to produce the three types of biocomposite:

(1) Five PLA sheets were alternatively stacked with four [0°/90°/90°/0°] orientated BW flax fabrics. The stack was preheated at 180 °C for two minutes using the heating plates. Then, the pressure was progressively increased to 16 MPa for three minutes maintaining the 180 °C. Finally, the composite was cooled at room temperature. The fiber–weight ratio was around 50.76%, and the resulting fiber volume fraction was 0.61 for each laminate, in which 0.32 corresponded to the warp direction and 0.29 to the weft direction. Furthermore, the percentage of crimp was 7.61% and 2.81% for the warp and weft yarns, respectively. Prior works provide a more thorough explanation of the manufacturing process [12,13,14].
(2) The UD/PLA biocomposite was fabricated by alternatively stacking six layers of PLA sheet and five layers of UD fabric in the order of [0°/90°/0°/90°/0°]. Then, the composites followed the same preheating process for two minutes as the previous BW/PLA biocomposite. Later, it went through the gradual compression step until reaching the target pressure of 0.25 MPa and a constant temperature of 180 °C. Different from BW/PLA fabrication, after this stage the UD/PLA was kept in the compression machine for another four minutes to ensure the uniformity of the composite. A high pressure applied to UD/PLA produced fiber misalignment, thus more manufacturing time was required. Finally, the fiber weight ratio of UD/PLA after cooling was around 27.2% and the resulting fiber volume fraction in each laminate was 0.36.
(3) The T/PLA biocomposite was composed of five layers of PLA sheet and four layers of UD fabric in [0°/90°/90°/0°] orientation. It followed the same drying, compressing, and cooling process as BW/PLA but the target pressure during compression was set to 8 MPa because the matrix was uniformly distributed with lower pressure than in the BW/PLA composite. Finally, the fiber–weight ratio of T/PLA was measured to be 42.79% and its fiber volume fraction was 0.1941 and 0.1705 for the warp and weft direction, respectively. Furthermore, the resulting percentage of crimp was 4.42% and 4.38% for the warp and weft yarns, respectively.

These manufacturing parameters were obtained after several optimization attempts at sample production. They aimed to obtain the maximum strength of each fabric type since each one has a particular yarn configuration, and the diffusion of the melted PLA would happen in a different way. Also, fabric misalignment could occur depending on the fabric–matrix–weight ratio for stitched UD fabric.

## 3. Experimental Setup

### 3.1. Tensile Test

The universal servohydraulic testing machine, Instron 8516 (Instron, Norwood, MA, USA), was employed for tensile test of BW/PLA, T/PLA, and UD/PLA samples with an effective gauge length of 50 mm and width of 20 mm. General configurations of the machine were displacement control at three constant strain rates of 2 × 10^−2^ s^−1^, 2 × 10^−3^ s^−1^, and 2 × 10^−4^ s^−1^ to analyze the strain rate sensitivity of the three flax/PLA composites. In addition, strains were measured using an Instron dynamic extensometer (2620-601).

On the other hand, UD/PLA composite was tested along the fiber direction, but the tensile behavior of BW/PLA and T/PLA samples were studied in warp and weft directions.

### 3.2. Drop-Weight Impact Test

The low-velocity impact tests were carried out in the drop-weight tower, Instron-Ceast Fractovist 6785, with an instrumented striker of 3.620 kg and a hemispherical nose of diameter equal to 20 mm. The lowest impact energy was 2 J because the damage was almost imperceptible, while highest impact energy was 24 J which perforated the samples.

Test samples were trimmed to square form of 80 × 80 mm^2^ and clamped in its four edges in a steel frame with a centered circular free area of 55 mm diameter for impact. The average thickness density of the samples is shown in Table 1.

### 3.3. Compression after Impact Test

**Figure 1:**
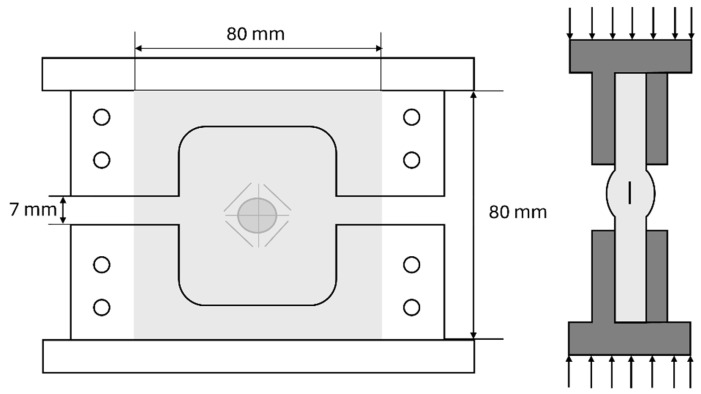
Compression after impact test configuration.

The compression after impact test of impacted samples from the drop-weight tower test helps evaluate the residual properties of the composites. The compression test configuration displayed in Figure 1, is an adapted version of those two CAI test fixtures used for the following standards: ASTM D7137 [15] and Boeing BSS 7260 [16]; and ISO 18352 [17] and Airbus AITM1-0010 [18]. The impact damage in the center area of the specimen could be exaggerated, easing its post-impact analysis of the composite strength, and this device could also avoid the global buckling occurrence of the specimen forcing thus the specimen to fail due to local buckling failure around the damaged zone [19,20]. The CAI test was evaluated using an Instron 8500 universal testing machine at a constant compression speed of 0.02 mm/s and a free space of 7 mm. Furthermore, four unimpacted samples were evaluated, as the references for the CAI comparative study.

## 4. Results and Discussion

### 4.1. Tensile Test

**Figure 2:**
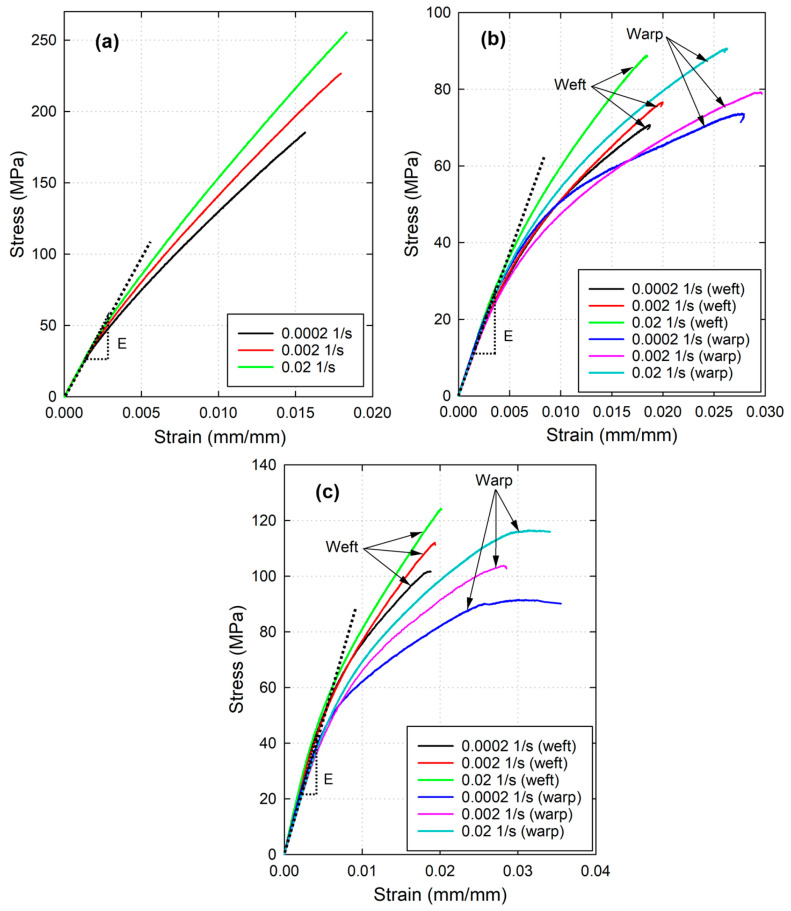
Stress–strain diagrams of (**a**) UD/PLA, (**b**) T/PLA, and (**c**) BW/PLA under three strain rate configurations.

Flax fiber-reinforced PLA composite behavior is strain rate dependent, thus uniaxial tensile tests were performed at three different strain rates (2 × 10^−2^ s^−1^, 2 × 10^−3^ s^−1^, and 2 × 10^−4^ s^−1^), six specimens were tested at each strain rate to verify the repeatability of the results. The maximum stress of the composite varies according to the strain rate, but the early elastic stage of loading always coincides. Therefore, it can be observed from Figure 2 and Table 2 that UD/PLA demonstrates the highest average initial modulus (E = 19.98 GPa), followed by BW/PLA, and T/PLA in the last place. For woven fabrics, the weft and warp yarns manifest different mechanical behaviors, therefore, both orientations were subjected to tensile tests. In fact, the modulus of the weft direction is slightly greater than the warp direction, i.e., the average values for BW/PLA are 10.01 GPa and 12.03 GPa in the warp and weft direction, respectively, even though the fiber volume fraction in the warp direction is slightly higher than the weft direction. The main reason that can explain this dissimilarity is that the warp direction presents a higher level of waviness (percentage of crimp), and it means that in this direction fabrics would present a higher degree of deformation under tensile loads than in the weft direction. Indeed, the micrographs have verified that the warp crimp is 7.61%, and the weft crimp is 2.81%.

On the other hand, the T/PLA biocomposite presents less variation in its warp and weft yarn directions (7.73 and 7.83 GPa, respectively), see Table 2. This similarity might be due to the weaving process of the twill 2-2 fabrics, which according to the technical data sheet of the manufacturer, the weight distribution is nearly the same for the warp and weft direction (51% and 49%, respectively). In fact, the micrographs demonstrated that the crimp percentages of the warp and weft yarn were highly similar (4.42% and 4.38%, respectively), and the volume fractions were comparable. Additionally, the warp yarn-orientated samples subjected to tensile test have always shown greater strain value than weft orientation in both BW/PLA and T/PLA samples.

Regarding the tensile strength of the biocomposites, BW/PLA exhibits higher tensile stress in the weft direction than in the warp direction under all strain rate cases. However, T/PLA presents almost the same stress level for every strain rate. Therefore, it can be deduced from the stress–strain diagram in Figure 2 that the T/PLA composite shows similar tensile strength and modulus regardless of the yarn orientation, but the deformation of the warp yarn is always larger than that of the weft yarn, which may also relate to the slightly higher volume fraction in the warp direction.

Comparing the above woven fabric-reinforced PLA composites with the UD/PLA, it can be seen that the latter has an average strain of 0.017 matchless with the warp-oriented of the former two, but comparable with the weft-orientated samples. This might be due to the less waviness of the unidirectional yarns in UD/PLA, which leads to lower deformation under tensile loads.

Furthermore, it is of great interest to see the presence of high linearity in the stress–strain diagrams of UD/PLA (Figure 2c). Natural fiber-reinforced polymer composites have a particular stress–strain diagram of undefined elastic stage and high nonlinearity up to rupture. Firstly, this early nonlinear stage is characterized by the progressive straightening of flax yarn under tensile loading, especially occurs in woven-fabric reinforced composite, causing thus possible fiber–matrix debonding phenomenon and nonlinear elastic response. At the microscopic level, the microfibril structural change also plays a significant role in this nonlinear behavior of the composite, according to the statement of Bourmaud et al. [21], Baley [22], and Charlet et al. [23]. Subsequently, for UD/PLA, as a response to the microfibril alignment, the unidirectional flax fiber drives the tensile behavior of the composite thus the tensile curve shows a linear tendency up to failure as the one of an elementary fiber. However, BW/PLA and T/PLA present more complex fiber–matrix and warp/weft yarn interactions that make them less linear, and therefore their tensile diagrams display a visco-plastic-like behavior.

Overall, the UD/PLA composite has demonstrated the highest tensile strength and initial modulus, then followed by BW/PLA and T/PLA. Regarding the strain at failure, BW/PLA experiences higher strains than T/PLA and UD/PLA.

### 4.2. Drop-Weight Impact Test

**Figure 3:**
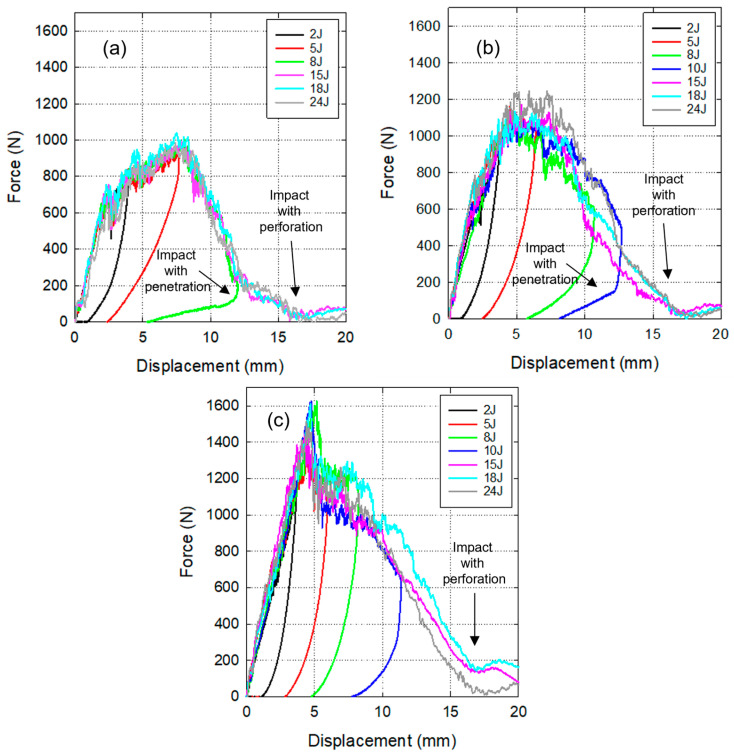
Force–displacement diagrams under different low-velocity impact energies, (**a**) UD/PLA, (**b**) T/PLA, and (**c**) BW/PLA.

Low-velocity impact tests were carried out for the three biocomposites, and the following results were obtained. The force–displacement curves of the biocomposites impacted from 2 J to 24 J are shown in Figure 3. Force–displacement curves can follow two different trends. For impacts lower than the limit energy (E_lim_), the force increases with displacement until the maximum value is reached, at this point, the striker rebounds leading to a reduction of displacement and contact force. In these cases, the final value of the force is zero, but the final displacement is not zero due to permanent deformations.

On the other hand, for impacts higher than E_lim_, displacement increases even when contact force decreases because there is a perforation of the laminate. For impact energy close to Elim, the sample suffers penetration, and its F-d diagram displays a transitional response between lower and higher impact energies. It can be deduced from Figure 3 that the Elim of UD/PLA is close to 8 J, and this threshold value is between 10 J and 15 J for twill and basket weave fiber-reinforced composites.

Regarding the maximum contact force, UD/PLA samples are in the range of 800 N to 1000 N, T/PLA samples are between 1000 N and 1200 N, and BW/PLA between 1200 N and 1600 N. It is evident that BW/PLA can undergo higher impact forces than the other two.

**Figure 4:**
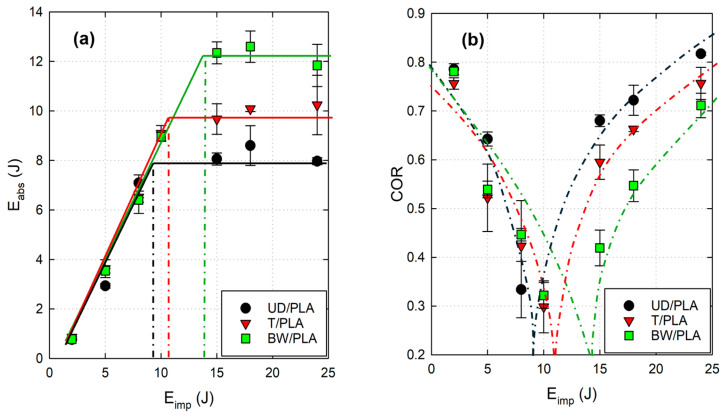
(**a**) Absorbed energy and (**b**) coefficient of restitution (COR) in terms of impact energies.

Absorbed energy (E_abs_) is calculated as the integral under the force–displacement curve. The absorbed energy as a function of impact energy is shown in Figure 4a for the three reinforcement configurations. For impact energy between 2 J and 8 J, there is a linear relationship between absorbed and impact energies, and the results are equal for the three biocomposites. Beyond these energies, E_abs_ starts to stabilize, and the upper limit that UD/PLA composite can withstand an impact without complete perforation seems to be around 9 J as presented in Figure 4a. BW/PLA is stabilizing around 14 J while the estimated E_lim_ of T/PLA is about 11 J. The observations from Figure 3 confirm these data.

Another impact performance parameter that also verifies the above outcomes is the coefficient of restitution (COR = [(E_imp_ − E_abs_)/E_imp_]^0.5) [20,24].

This parameter implies the transition moment (see Figure 4b); as expected, the coefficient decreases as the sample perforation gets closer, and COR increases afterward. Therefore, the point where COR is null shows the estimated E_lim_ of the biocomposite.

The BW/PLA samples exhibit the highest energy absorption capacity as expected, and it is attributed to the higher fiber–weight ratio of the composite. Another potential reason might be due to the high percentage of fiber crimp of the basket weave fabric that gives it greater deformability under loading.

**Figure 5:**
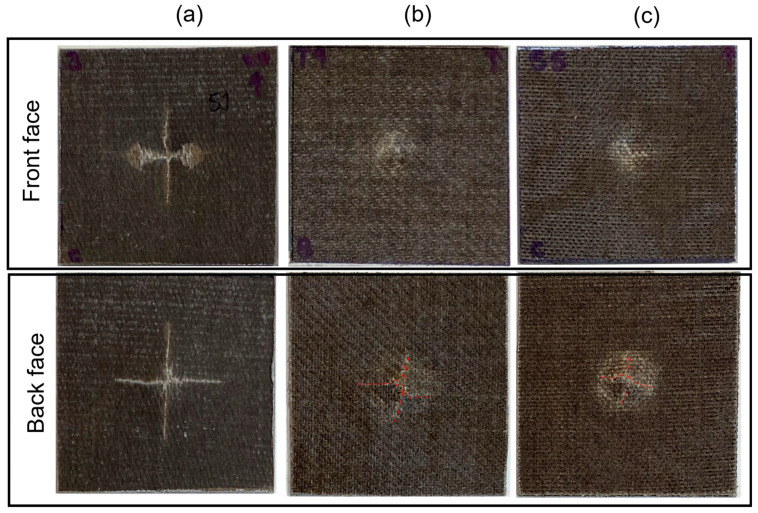
Front and back face damage of (**a**) UD/PLA, (**b**) T/PLA, and (**c**) BW/PLA at 5 J of impact energy.

**Figure 6:**
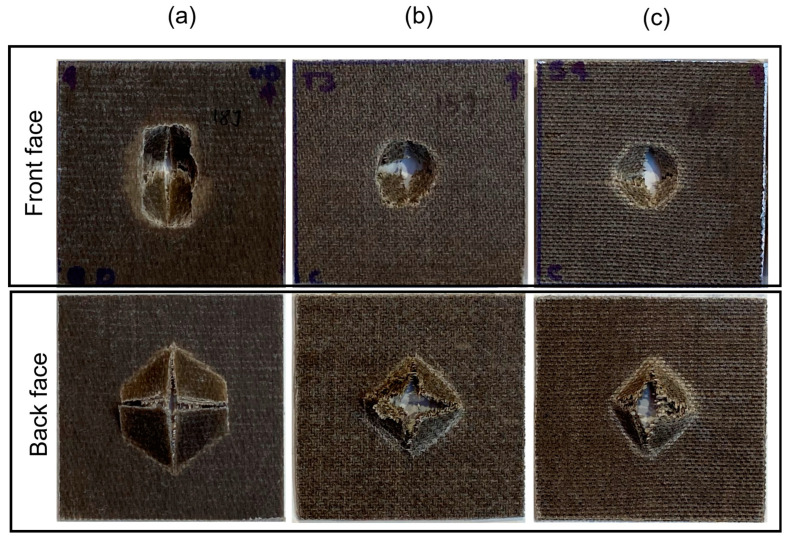
Front and back face damage of (**a**) UD/PLA, (**b**) T/PLA, and (**c**) BW/PLA at 15 J of impact energy.

The impact damage area of the front and back sides for all cases considered are shown in Figure 5 and Figure 6 for impact energies of 5 J and 15 J, respectively.

**Figure 7:**
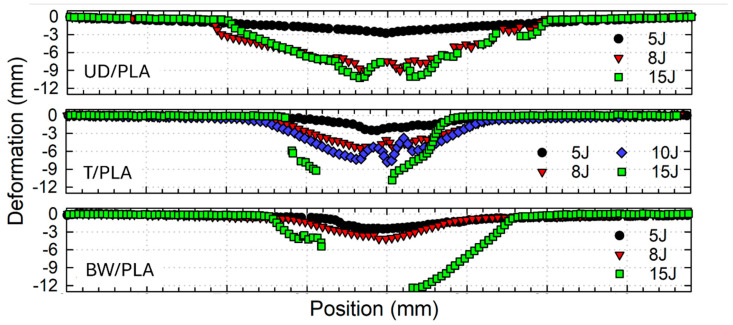
Back-face permanent deformation of UD/PLA, T/PLA, and BW/PLA after drop-weight impact test by profilometer.

At 5 J of incident energy, Figure 5a, both the front and back sides of UD/PLA present a cross-shaped crack; in comparison, the front crack shows a more prolonged fissure along the longitudinal fiber direction, which is reasonable due to the load transmission. The damage mode of 2 J is similar but less pronounced than 5 J, although this damage resulted in permanent deformation, this was essentially due to the localized damage in the contact area. However, beyond this impact energy, the permanent deformation area is sharper as represented in Figure 7, where the profiles of permanent deformations are shown. For impact energy of 15 J, see Figure 6a, the cross-shaped crack in the back side is surrounded by a unique hexagon-shaped fracture. This petalling effect has persisted for further impact energies and might be caused by the cross-ply stacking sequence of the present composite ([0°/90°/0°/90°/0°]) which is distinct from entirely 0° orientated UD composites such as those damaged areas observed in the works conducted by Baysal et al. [25] and Lawrence et al. [26].

On the other hand, T/PLA and BW/PLA both have similar annular cracks in the front and back sides at 5 J (Figure 5b,c), and a small cross-shaped fissure is presented in the back face that implies fiber rupture and matrix cracking phenomena of the composite due to bending force at this impact energy level. When increasing energy, the perforation hole in the frontal face is evident for both cases considered; the petalling effect in the back surface is considerably enlarged and interlayer debonding is clearly detected by visual inspection around the impact zone, as shown in Figure 6b,c.

Overall, the damage area in UD/PLA was larger than in T/PLA and BW/PLA for both 5 J and 15 J of impact energies. This observation is reasonable since unidirectional fiber has a higher tendency to damage propagation in its longitudinal direction than the woven samples.

Figure 7 represents the permanent deformation of impacted samples at increasing energies. As can be seen, the deformation evolution is directly related to the impact energy, and it results in the back petal size of the sample. This phenomenon is similar for all three types, but the bending area is wider for the UD/PLA composite at all impact energies than BW/PLA, and T/PLA presents the most minor bending area between them.

### 4.3. CAI Test

**Figure 8:**
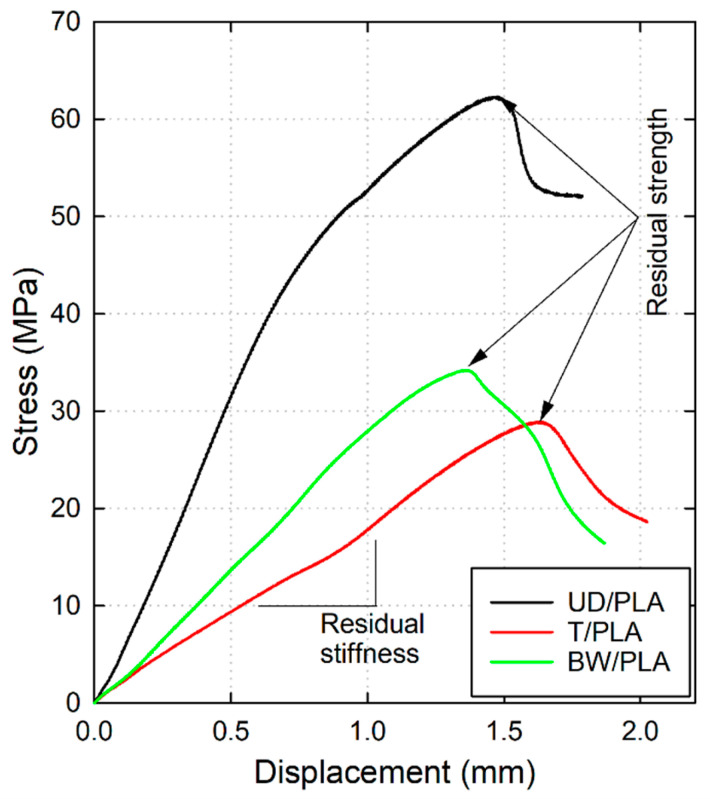
CAI force–displacement of UD/PLA, T/PLA, and BW/PLA unimpacted samples.

The result from the CAI test shows the growth of the compression stress of the sample with the displacement until it reaches the compressive failure, and then the stress sharply drops. Two essential parameters can be obtained from the test: the residual strength and the residual stiffness. The first one represents the maximum stress that the sample could endure during the test, and the stiffness indicates the slope of the CAI stress–displacement diagram, as shown in Figure 8.

**Figure 9:**
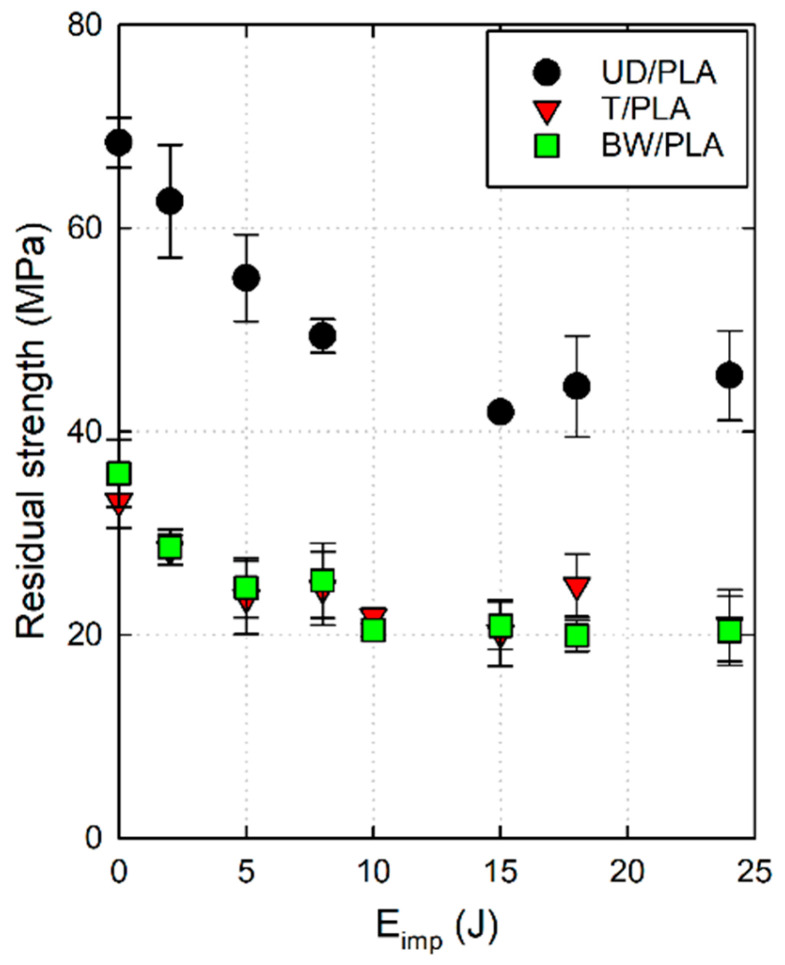
CAI residual strength versus impact energy of the three composite samples.

Figure 9 shows the residual strength of the biocomposites after the drop-weight impact test represented in a scatter plot. As can be seen, the three types of biocomposite manifest a similar tendency, gradual decline of the resistance as the impact energy increases until 15 J. Generally, the UD/PLA samples showed the greatest CAI residual strengths between the three composites under all impact energies. More specifically, for unimpacted samples (0 J) the average residual strength of UD/PLA is 68.42 MPa followed by BW/PLA and T/PLA, 35.88 MPa and 33.14 MPa, respectively. Furthermore, BW/PLA and T/PLA exhibit similar levels of residual strength for any impact energies except at 18 J. As observed from the above section, it is known that the threshold energy when all samples were perforated by the impactor during the low-velocity impact test was to be around 15 J, therefore, a comparison between unimpacted specimen’s CAI residual strength and that of specimens at 15 J was carried out and it reveals that BW/PLA has decreased by 41.80% of its CAI residual strength and T/PLA by 39.11%, while UD/PLA presents an average decrease in its compression strength by 38.73%. In summary, although the overall residual compressive strength of UD/PLA is more than twice as high as the other two, the CAI residual strength of the three has been reduced to the same percentage (~−40%) compared with the unimpacted sample.

**Figure 10:**
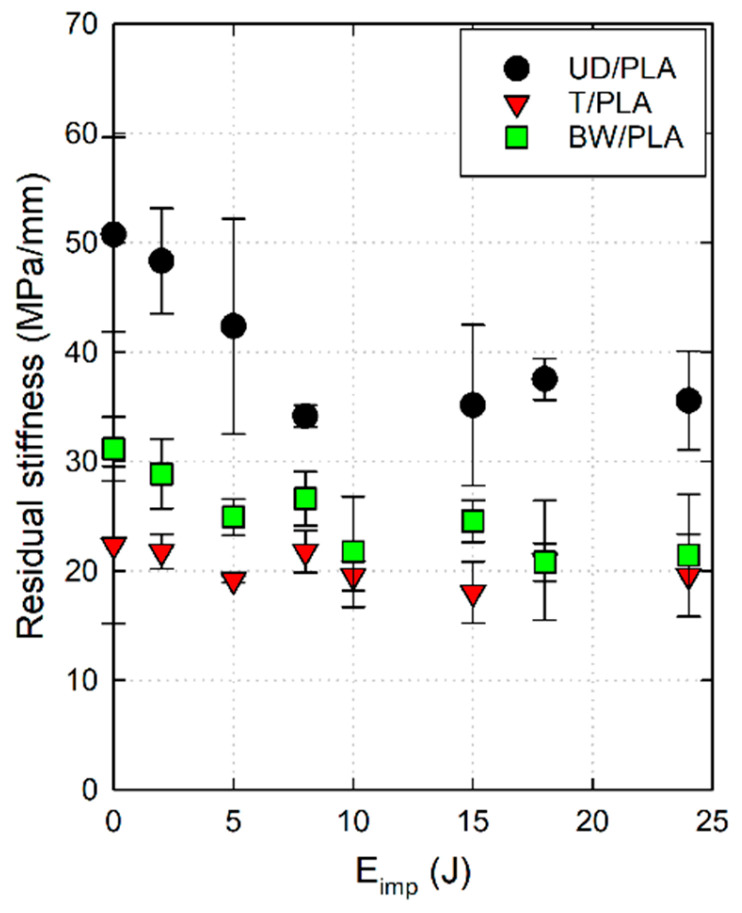
CAI residual stiffness versus impact energy of the three composite samples.

Figure 10 is also a scatter plot that represents the residual stiffness of the three composite samples subjected to different impact energies. In this case, the decrease in the compression stiffness is less pronounced than the CAI residual strength, however, values show an important reduction of the composite’s properties compared with the reference one, i.e., the stiffness reductions at 15 J were 30.75% for UD/PLA, 19.41% for T/PLA, and 21.28% for BW/PLA. Concisely, unlike the results in which the residual compressive strength of the above three composites is reduced to the same extent, in terms of residual compressive stiffness, the UD/PLA composite is more affected by low-velocity impact damage relative to the other two. The reason might be the greater damage area of UD/PLA after impact, which severely harms its stiffness, according to the pictures shown in Figure 5 and Figure 6.

## 5. Conclusions

The present work has comparatively studied the tensile response, impact behavior, and residual compression performance of unidirectional, basket weave, and twill weave flax/PLA biocomposites manufactured by the compression molding method. The following conclusions are drawn from the analysis:

- Due to lower fiber crimp, the UD/PLA composite exhibits the highest tensile strength and initial modulus than BW/PLA and T/PLA. On the other hand, BW/PLA experiences the highest strains.
- The low-velocity impact tests show that BW/PLA samples have the highest energy absorption capability due to the composite highest fiber–weight ratio. The high fiber crimp of basket weave fabric can also explain the best impact response.
- The impacted damage area of the UD/PLA composite is higher than that of T/PLA and BW/PLA due to its internal architecture for both pre-perforation and post-perforation energies.
- Regarding the CAI results, the residual compressive strength of all three composites was reduced by 40% compared to the reference sample. The stiffness reduction was different for each reinforcement architecture, UD/PLA composite, with a larger damage area after impact, which seriously affects its stiffness, and shows a reduction of 30%, while the reduction in BW/PLA and T/PLA composites was about 20%.

Finally, these findings have significant implications for the practical use of the studied NFRP composites and highlight potential areas for improvement in their application. Previous studies showed how green composites have a great energy absorption capability, the present work shows how this potential can be improved by the proper reinforcement configuration.

## Figures and Tables

**Table 1:** Average areal density and thickness of the composites.

Composite	Areal Density (g/m^2^)	Thickness (mm)
UD/PLA	2640.6	2.03
T/PLA	2703.1	2.68
BW/PLA	2860.9	2.57

**Table 2:** Mechanical properties of UD/PLA, T/PLA, and BW/PLA composites including initial modulus, tensile strength, and ultimate strain.

		Initial Modulus (GPa)	Tensile Strength (MPa)			Ultimate Strain (-)		
			2 × 10^−2^ s^−1^	2 × 10^−3^ s^−1^	2 × 10^−4^ s^−1^	2 × 10^−2^ s^−1^	2 × 10^−3^ s^−1^	2 × 10^−4^ s^−1^
UD/PLA		19.98	255.36	226.67	185.36	0.018	0.018	0.016
T/PLA	Warp	7.73	90.67	79.28	73.72	0.026	0.030	0.028
	Weft	7.83	88.81	76.66	88.81	0.018	0.020	0.018
BW/PLA	Warp	10.01	116.54	103.84	91.58	0.034	0.029	0.036
	Weft	12.03	124.21	112.09	101.76	0.020	0.019	0.019

## Data Availability

The raw/processed data required to reproduce these findings cannot be shared at this time as the data also forms part of an ongoing study.
